# Prevalence and Impact of Malnutrition Risk on Outcomes in Critically Ill Patients with Traumatic Brain Injury and Stroke: A Retrospective Cohort Study Using Electronic Health Records

**DOI:** 10.3390/nu16152396

**Published:** 2024-07-24

**Authors:** Alexander E. Shestopalov, Alexandra V. Yakovleva, Mikhail Ya. Yadgarov, Ivan V. Sergeev, Artem N. Kuzovlev

**Affiliations:** 1 Federal Research and Clinical Center of Intensive Care Medicine and Rehabilitology, Moscow 107031, Russia; ashest@yandex.ru (A.E.S.); avyakovleva@fnkcrr.ru (A.V.Y.); dr.1vansergeev@yandex.ru (I.V.S.); artem_kuzovlev@fnkcrr.ru (A.N.K.); 2 V. M. Gorbatov Federal Research Center for Food Systems, Russian Academy of Sciences, Moscow 109316, Russia

**Keywords:** malnutrition, ICU, stroke, traumatic brain injury, prognostic nutritional index

## Abstract

Background: Malnutrition is a critical concern in ICU settings. It is associated with increased morbidity and mortality, yet its prevalence and impact on clinical outcomes in patients with stroke and traumatic brain injury (TBI) remain underexplored. Objective: To evaluate the prevalence and impact of malnutrition risk on clinical outcomes in ICU patients with TBI, ischemic stroke, and hemorrhagic stroke, and to identify key risk factors associated with malnutrition risk. Methods: This retrospective cohort study utilized electronic health records encompassing ICU admissions from 2017 to 2023. Patients with either stroke or TBI were included, with malnutrition risk assessed using the prognostic nutritional index. Data were extracted and analyzed to determine patient characteristics, clinical and laboratory parameters, and outcomes. Results: This study included 1352 patients (267 TBI, 825 ischemic stroke, and 260 hemorrhagic stroke patients, >30% with pneumonia at admission). Severe malnutrition risk at admission was observed in over 60% of patients. Stroke patients, particularly those with hemorrhagic stroke, exhibited a higher risk of malnutrition compared to TBI patients. Malnutrition risk was associated with significantly higher hospital mortality and increased need for mechanical ventilation. Predictive factors for malnutrition risk included advanced age, higher SOFA scores, lower FOUR and GCS scores, and the presence of pneumonia at admission. Conclusions: Risk of malnutrition is highly prevalent among ICU patients with TBI, ischemic, and hemorrhagic stroke, significantly impacting mortality and other clinical outcomes. Identifying and managing malnutrition early in the ICU setting is crucial for improving patient outcomes. Further prospective, multicenter studies are needed to validate these findings and develop effective interventions.

## 1. Introduction

“Brain damage” or “brain injury” refers to a severe acute condition affecting the central nervous system, arising from various causes but sharing common pathogenic mechanisms that lead to similar neuropathological syndromes. Stroke and traumatic brain injury are among the most prominent and leading types of brain damage [1].

Among all diagnosed acute cerebrovascular events, ischemic strokes account for 65–85%. Although less frequent, hemorrhagic strokes carry a higher mortality rate [2], ranging from 30 to 50%, with only 10–20% of patients recovering functional activity [3]. Even with the best available treatment, over 30% of stroke survivors are left with severe disabilities, and 20% require specialized medical care three months post-stroke [4]. Of those who survive, 60% become disabled yet manage self-care, 19–35% are entirely dependent on others, and only 15–20% resume their professional lives. Long-term disability is the most prevalent post-stroke complication, posing a significant socioeconomic burden [4,5]. Similarly, traumatic brain injury (TBI) remains a critical medical and public health issue worldwide. TBI is the foremost cause of death and disability among young and middle-aged adults, the most productive and socially active segment of the population. The average mortality rate for severe TBI is a staggering 39%, with 60% of patients experiencing unfavorable outcomes as measured by the Glasgow Outcome Score. TBI holds the grim distinction of being the leading cause of mortality and disability for individuals under 44 years of age [6].

Severe brain injuries result not only in profound physical and mental impairments but also in significant disruptions to metabolism and nutritional status. Malnutrition in patients with brain injuries is a critical concern due to its widespread prevalence, including among young and middle-aged individuals. This condition often leads to serious complications such as infectious and trophic disorders. Patients frequently develop issues like pneumonia, bedsores, and polyneuropathy, which are associated with prolonged immobilization and impaired nutritional status [7,8,9]. Additionally, the high rates of mortality and disability, along with the severe and prolonged loss of work capability, underscore the gravity of these consequences [10,11,12]. 

Malnutrition and metabolic disturbances are among the foremost complications in stroke patients, significantly impacting the prognosis and treatment outcomes [13,14]. Expert assessments indicate that approximately 20% of patients admitted with acute stroke suffer from malnutrition, with some studies reporting figures as high as 62%. Three weeks post-admission, 56.3% of patients with severe stroke exhibit signs of malnutrition. By the time of discharge, 61% of stroke patients are found to be at risk of malnutrition [15,16,17].

The prevalence and impact of malnutrition on outcomes in critically ill patients with TBI and stroke is a multifaceted issue. Despite extensive data on the nutritional status, protein, and energy deficiencies in these patients, there remains a lack of clarity regarding the true incidence of malnutrition in these populations. 

Although the ESPEN guidelines on clinical nutrition in neurology [18] indicate a strong consensus on the importance of screening for malnutrition at admission and provide comprehensive methods for early diagnosis, prevention, and correction of nutritional deficiencies and metabolic disorders, there remains variability in their implementation across clinical settings. This inconsistency may hinder the optimization of treatment outcomes and the efficacy of rehabilitation efforts. 

The aim of this study was to evaluate the prevalence, outcomes, and predictors of malnutrition risk among ICU patients with traumatic brain injury, ischemic stroke, and hemorrhagic stroke.

## 2. Materials and Methods

### 2.1. Data Sources

This retrospective, single-center cohort study utilized electronic health records (EHRs) from the Federal Research and Clinical Center of Intensive Care Medicine and Rehabilitology (Russian Intensive Care Dataset, RICD [19,20]). The data encompassed all patients admitted to the intensive care units (ICUs) between December 2017 and July 2023. The study was approved by the local ethics committee (No. 4/23/2, 20 December 2023).

### 2.2. Selection Criteria

This study included patients diagnosed with either stroke or TBI who were admitted to the ICU. The exclusion criteria were as follows: (1) nonprimary ICU admission in our medical center, (2) length of ICU stay <24 h, (3) anoxic brain injury, (4) brain tumors. The patients with missing information on malnutrition risk status at admission and ICD-10 diagnoses were also excluded. The risk of malnutrition was assessed at ICU admission using prognostic nutritional index (PNI) score, and PNI was calculated as [10 × serum albumin (g/dL)] + [0.005 × absolute lymphocyte count (per mm^3^)] [21]. Patients were stratified into three nutritional status groups based on PNI scores: normal nutrition (PNI > 38), moderate risk of malnutrition (PNI = 35–38), and severe risk of malnutrition (PNI < 35) [22]. We categorized moderate and severe in the same group “at risk of malnutrition”.

### 2.3. Data Extraction

The data from EHRs were extracted using SQLite version 3.45.2 (https://www.sqlite.org/, accessed on 14 June 2024). The parameters analyzed included the following: (1) general patient information-sex, age, body mass index (BMI), and whether the patient was transferred from another hospital; (2) clinical data-acute physiology and chronic health evaluation II (APACHE II), sequential organ failure assessment (SOFA), Full Outline of UnResponsiveness (FOUR), Glasgow coma scale (GCS), and Coma Recovery Scale-Revised (CRS-R) scores at admission, pneumonia at admission, and comorbidity; (3) laboratory data at admission for white blood cell (WBC) count, neutrophil and lymphocyte counts, neutrophil-to-lymphocyte ratio (NLR), platelet count, International Normalized Ratio (INR), albumin, total protein, and cholesterol; and (4) outcomes of hospital mortality, length of hospital stay, need for mechanical ventilation (MV), use of vasoactive drugs, and discharge location. For patients with multiple laboratory measurements on the same day, the earliest values were analyzed. 

### 2.4. Outcomes

The primary endpoint was the prevalence of malnutrition risk among ICU patients with TBI, ischemic, and hemorrhagic strokes. The secondary endpoints included all-cause mortality, MV and vasoactive drugs usage, and the duration of hospitalization. Additional analyses identified risk factors associated with the malnutrition risk. 

### 2.5. Statistical Analysis

Data distribution was assessed using the Shapiro–Wilk test. Continuous variables were reported as medians (Me) and interquartile ranges (IQRs), while categorical variables were expressed as frequencies and percentages. The chi-square test and Fisher’s exact test (with Fisher–Freeman–Halton exact extension where applicable) were used for comparing categorical variables. The Kruskal–Wallis test and Mann–Whitney U-test (Wilcoxon rank-sum test) were applied for continuous variables.

Survival analysis was performed using Kaplan–Meier survival curves, with cumulative risk functions estimated via the Nelson–Aalen estimator. Differences in survival between groups were evaluated using the log-rank test. Multivariable Cox regression analysis was conducted to identify independent predictors of mortality, with backward feature selection (Wald) revealing adjusted hazard ratios (HRs) and 95% confidence intervals (CIs).

The optimal cutoff point for the PNI score was determined using receiver operating characteristic (ROC) analysis and the Youden index to maximize sensitivity and specificity. Missing data were not imputed. Statistical significance was set at *p* < 0.05 (two-sided) if a Bonferroni multiple-significance-test correction was not applied. All the statistical calculations were performed using IBM SPSS Statistics v. 29.0 and Stata v. 18.0.

## 3. Results

### 3.1. Patient Characteristics

**Figure 1:**
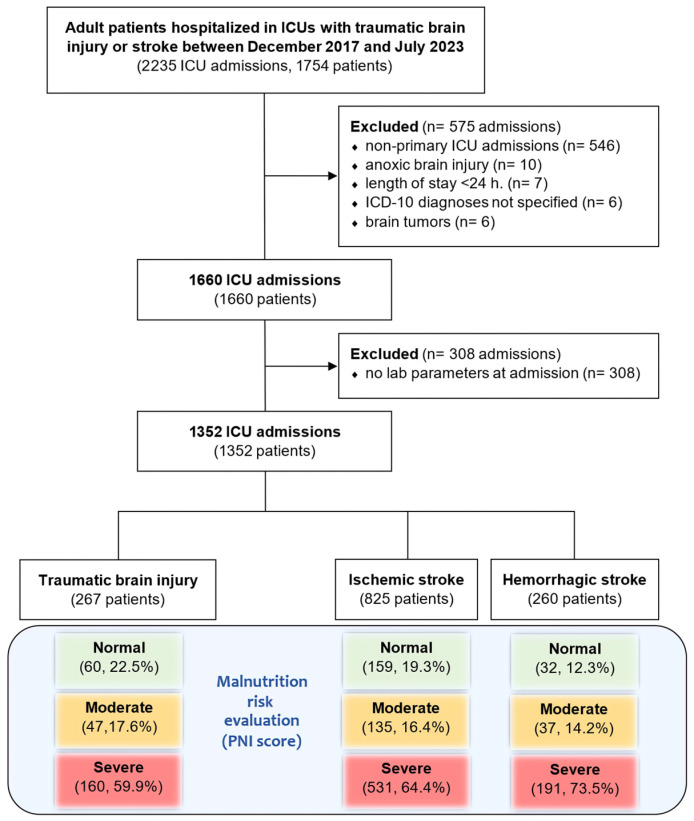
Flowchart of patient selection in the study.

A flowchart depicting the patient selection process for the study is presented in Figure 1. Initially, 1754 patients with either stroke or TBI were admitted to the ICUs. Following the application of exclusion criteria, 1352 patients were included in the final analysis. This cohort comprised 267 patients with TBI (20%), 825 patients with ischemic stroke (61%), and 260 patients with hemorrhagic stroke (19%).

Baseline characteristics, disease characteristics, and outcomes for all patients are summarized in Table 1. The mean age was 42 years (IQR 29–56) in the TBI group, 69 years (IQR 60–78) in the ischemic stroke group, and 59 years (IQR 48–68) in the hemorrhagic stroke group (*p* < 0.001). The TBI group had a predominantly male population, whereas females were more prevalent in both stroke groups. Over 90% of stroke patients and 64% of TBI patients were transferred from other hospitals. Stroke patients exhibited a higher prevalence of comorbidities.

Severe risk of malnutrition at admission was observed in over 60% of the patients. Hospital mortality rates were 6.7% for the TBI group, 8.1% for the hemorrhagic stroke group, and 14.8% for the ischemic stroke group (*p* < 0.001). Mechanical ventilation was required for more than 40% of the patients, and vasoactive drugs were administered to 13–18% of patients, as detailed in Table 1. Patients with TBI had a longer median hospital stay (39 days) compared to those with hemorrhagic stroke (36 days) and ischemic stroke (31 days), *p* < 0.001. Approximately half of the patients were discharged to other ICUs, while the remainder were discharged for further treatment or rehabilitation.

A Strengthening the Reporting of Observational Studies in Epidemiology (STROBE) checklist for this study is provided in Supplementary Material Table S1.

### 3.2. Prevalence of Malnutrition Risk

A significant proportion of patients exhibited severe risk of malnutrition upon ICU admission, with over 60% of those with TBI and ischemic stroke, and over 70% of patients with hemorrhagic stroke (Table 1). Specifically, the PNI scores were lowest among hemorrhagic stroke patients, with a median of 31.3 (IQR 27.9–35.3), followed by ischemic stroke patients with a median of 32.2 (IQR 27.3–36.7), and TBI patients with a median of 33.4 (IQR 28.2–37.7) (*p* = 0.025).

Stroke patients exhibited significantly elevated NLR values (*p* < 0.001) and reduced levels of platelets (*p* < 0.001), INR (*p* = 0.026), albumin (*p* = 0.025), total protein (*p* < 0.001), and cholesterol (*p* = 0.009) (Table 1).

### 3.3. Outcomes

**Figure 2:**
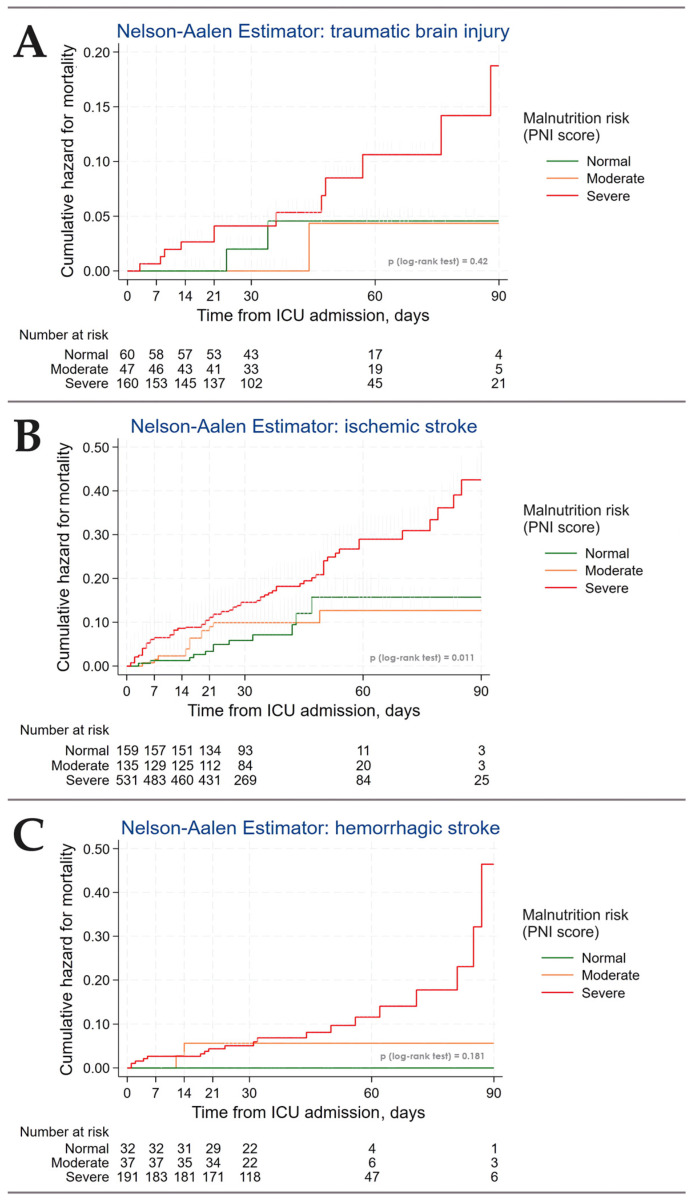
Cumulative hospital mortality risk stratified by malnutrition risk status according to PNI score. (**A**) Traumatic brain injury, (**B**) ischemic stroke, (**C**) hemorrhagic stroke.

The analysis of cumulative hospital mortality risk revealed a significantly increased risk for patients with ischemic stroke and moderate/severe risk of malnutrition compared to those with normal nutritional status (*p* = 0.011, Figure 2).

Patients at risk of malnutrition exhibited significantly higher hospital mortality rates in the TBI (9.9% vs. 3.2%, *p* = 0.048) and ischemic stroke (22.8% vs. 9.0%, *p* < 0.001) groups, whereas no significant difference was observed in the hemorrhagic stroke group (*p* = 0.181) (Table 2 and Table 3). PNI score was a significant predictor of mortality in univariate analysis (HR 0.918, *p* < 0.001), but only age and SOFA remained significant predictors in multivariable analysis (Table 2).

Optimal cutoff points for the PNI score to predict mortality were established for each patient group: <33.8 for TBI, <31.6 for hemorrhagic stroke, and <30.8 for ischemic stroke (Table 3). Based on these thresholds, malnutrition risk was identified in 142 out of 267 (53%) TBI patients, 347 out of 825 (42%) ischemic stroke patients, and 136 out of 260 (52%) hemorrhagic stroke patients.

Additionally, malnutrition risk was associated with an increased need for MV across all patient groups (*p* < 0.001) and a higher requirement for vasoactive drugs, except in the hemorrhagic stroke group. Notably, ischemic stroke patients at risk of malnutrition also had shorter hospital stays (Table 3).

### 3.4. Predictors of Malnutrition Risk

Patients with stroke, particularly those with hemorrhagic stroke, exhibited a higher risk of malnutrition compared to patients with TBI (Table 1).

In all groups, patients at risk of malnutrition were generally older (*p* < 0.001), had higher SOFA scores, lower FOUR, GCS scores (*p* < 0.001), and CRS-R scores (*p* = 0.037), and were more likely to have pneumonia at admission (*p* < 0.01) (Table 3). Female sex emerged as a statistically significant risk factor among patients with hemorrhagic stroke (*p* = 0.004). Coronary artery disease was identified as a risk factor for malnutrition across all patient groups (*p* < 0.01), while arterial hypertension was specifically significant in the TBI group (*p* < 0.001).

Patients at risk of malnutrition demonstrated elevated WBC counts, NLR, and INR, along with reduced levels of albumin, total protein, and cholesterol (all *p* < 0.03). Additionally, platelet counts were significantly lower in patients with ischemic stroke (*p* < 0.001). Moreover, patients with elevated malnutrition risk and ischemic stroke also presented with a higher prevalence of anemia (*p* = 0.04) (Table 3).

**Table 3:** Outcomes and predictors of malnutrition risk.

Parameters		Traumatic Brain Injury, *n* = 267			Ischemic Stroke, *n* = 825			Hemorrhagic Stroke, *n* = 260		
		Normal Nutrition (Not at Risk of Malnutrition) *n* = 125 PNI ≥ 33.8 ^†^	At Risk of Malnutrition *n* = 142 PNI < 33.8 ^†^	*p* Value	Normal Nutrition (Not at Risk of Malnutrition) *n* = 478 PNI ≥ 30.8 ^†^	At Risk of Malnutrition *n* = 347 PNI < 30.8 ^†^	*p* Value	Normal Nutrition (Not at Risk of Malnutrition) *n* = 124 PNI ≥ 31.6 ^†^	At Risk of Malnutrition *n* = 136 PNI < 31.6 ^†^	*p* Value
Sex	male	85, 68%	108, 76%	0.142 ^1^	274, 57%	175, 50%	0.05 ^1^	82, 66%	66, 49%	0.004 ^1^
	female	40, 32%	34, 24%		204, 43%	172, 50%		42, 34%	70, 51%	
Age, years		34 (IQR 24–48)	48 (IQR 37–60)	<0.001 ^2^	66 (IQR 58–75)	72 (IQR 63–81)	<0.001 ^2^	52.5 (IQR 41–63.5)	63 (IQR 56–69.5)	<0.001 ^2^
BMI, kg/m^2^		* *n* = 113, 21.6 (IQR 19.0–24.6)	*n* = 127, 22.1 (IQR 18.9–25.4)	0.4 ^2^	*n* = 425, 26.1 (IQR 23.0–30.5)	*n* = 319, 26.1 (IQR 22.8–30.8)	0.8 ^2^	*n* = 118, 25.6 (IQR 21.8–29.4)	*n* = 122, 25.5 (IQR 22.6–30.03)	0.8^6^
SOFA at admission, score		*n* = 78, 2 (IQR 1–3)	*n* = 104, 3 (IQR 2–5)	<0.001 ^2^	*n* = 330, 2 (IQR 1–3)	*n* = 269, 4 (IQR 3–6)	<0.001 ^2^	*n* = 89, 2 (IQR 0–3)	*n* = 92, 3 (IQR 2–4)	<0.001 ^2^
FOUR at admission, score		*n* = 79, 15 (IQR 14–16)	*n* = 110, 13 (IQR 10–15)	<0.001 ^2^	*n* = 352, 16 (IQR 15–16)	*n* = 269, 13 (IQR 9–16)	<0.001 ^2^	*n* = 97, 16 (IQR 14–16)	*n* = 94, 13 (IQR 11–16)	<0.001 ^2^
GCS at admission, score		*n* = 81, 13 (IQR 10–15)	*n* = 111, 10 (IQR 8–14)	<0.001 ^2^	*n* = 358, 15 (IQR 12–15)	*n* = 277, 11 (IQR 8–14)	<0.001 ^2^	*n* = 98, 14 (IQR 12–15)	*n* = 97, 11 (IQR 9–14)	<0.001 ^2^
CRS-R at admission, score		*n* = 59, 14 (IQR 5–22)	*n* = 69, 10 (IQR 5–18)	0.037 ^2^	*n* = 228, 22 (IQR 17–23)	*n* = 165, 13 (IQR 5–20)	<0.001 ^2^	*n* = 54, 21 (IQR 17–22)	*n* = 67, 12 (IQR 5–20)	<0.001 ^2^
Pneumonia at admission		31, 25%	57, 40%	0.008 ^1^	108, 23%	184, 53%	<0.001 ^1^	24, 20%	65, 48%	<0.001 ^1^
Coronary artery disease		2, 1.6%	15, 10.6%	0.002 ^3^	88, 19%	125, 36%	<0.001 ^1^	13, 11%	31, 23%	0.008 ^1^
Arterial hypertension		23, 18%	59, 42%	<0.001 ^1^	376, 79%	257, 74%	0.123 ^1^	92, 74%	105, 77%	0.6 ^1^
Type 2 diabetes		0, 0%	3, 2.1%	0.3 ^3^	26, 5.4%	26, 7.5%	0.2 ^3^	6, 4.8%	5, 3.7%	0.7 ^3^
Anemia		5, 4.0%	12, 8.5%	0.2 ^3^	7, 1.5%	13, 3.7%	0.04 ^3^	3, 2.4%	10, 7.4%	0.089 ^3^
WBC, 10^9^/L		7.4 (IQR 6.2–9.7)	9.1 (IQR 7.1–11.5)	0.002 ^2^	8.3 (IQR 6.6–10.6)	9.83 (IQR 7.3–12.6)	<0.001 ^2^	8.5 (IQR 6.45–11.575)	8.7 (IQR 6.6–11.62)	0.6^6^
NLR		3.2 (IQR 2.2–5.3)	5.0 (IQR 3.2–8.2)	<0.001 ^2^	3.9 (IQR 2.6–6.6)	7.1 (IQR 4.2–12.6)	<0.001 ^2^	3.5 (IQR 2.5–7.4)	6 (IQR 3.9–10.0)	<0.001 ^2^
Platelets, 10^9^/L		344 (IQR 260–442)	325 (IQR 244–416)	0.3 ^2^	264 (IQR 212–332)	242 (IQR 185–314)	<0.001 ^2^	311 (IQR 231–379.5)	294 (IQR 226–376.5)	0.5 ^2^
INR		*n* = 123, 1.14 (IQR 1.06–1.27)	*n* = 139, 1.21 (IQR 1.13–1.36)	0.004 ^2^	*n* = 474, 1.11 (IQR 1.02–1.24)	*n* = 342, 1.225 (IQR 1.1–1.35)	<0.001 ^2^	*n* = 123, 1.12 (IQR 1.06–1.22)	*n* = 135, 1.2 (IQR 1.08–1.35)	0.001 ^2^
Albumin, g/L		37.9 (IQR 35.9–40.7)	28.35 (IQR 25.1–31.9)	<0.001 ^2^	36 (IQR 33.4–38.8)	26.5 (IQR 23.8–28.7)	<0.001 ^2^	35.85 (IQR 33.55–38.15)	28 (IQR 25.55–30)	<0.001 ^2^
Total protein, g/L		*n* = 124, 68.8 (IQR 66.4–72.2)	*n* = 140, 59.2 (IQR 54.5–64.9)	<0.001 ^2^	*n* = 460, 66.2 (IQR 62.2–70.2)	*n* = 335, 55.4 (IQR 50.7–59.4)	<0.001 ^2^	*n* = 119, 65.7 (IQR 62.6–70.1)	*n* = 135, 57 (IQR 53.2–61.6)	<0.001 ^2^
Cholesterol, mmol/L		*n* = 12, 5.71 (IQR 4.52–6.37)	*n* = 4, 3.79 (IQR 3.13–4.52)	0.02 ^2^	*n* = 39, 4.42 (IQR 3.62–5.43)	*n* = 25, 3.37 (IQR 2.92–3.82)	0.002 ^2^	*n* = 10, 5.46 (IQR 4.43–6.02)	*n* = 13, 3.61 (IQR 2.88–4.38)	0.004 ^2^
Outcomes										
Hospital mortality		4, 3.2%	14, 9.9%	0.048 ^3^	43, 9.0%	79, 22.8%	<0.001 ^3^	7, 5.6%	14, 10.3%	0.181 ^3^
Hospital length of stay, days		38 (IQR 27–62)	39.5 (IQR 22–64)	0.9 ^2^	34 (IQR 23–47)	25 (IQR 22–47)	<0.001 ^2^	36.5 (IQR 26.5–52)	35 (IQR 23–60)	0.6 ^2^
Need for MV		42, 34%	84, 59%	<0.001 ^1^	123, 26%	237, 68%	<0.001 ^1^	47, 38%	90, 66%	<0.001 ^1^
Use of vasoactive drugs		12, 9.6%	32, 23%	0.005 ^2^	52, 11%	92, 27%	<0.001 ^1^	11, 8.9%	23, 16.9%	0.066 ^2^

1—Chi-square test; 2—Mann–Whitney U-test; 3—Fisher’s exact test (Fisher–Freeman–Halton exact test). Qualitative data presented in Me (IQR) format. * Number of patients (*n*) is given when missing data are present. ^†^ Optimal cut-off values from ROC-analysis. Abbreviations: BMI, body mass index; SOFA, Sequential Organ Failure Assessment; FOUR, Full Outline of UnResponsiveness; GCS, Glasgow coma scale; CRS-R, Coma Recovery Scale-Revised; MV, mechanical ventilation; PNI, prognostic nutritional index; WBC, white blood cell count; NLR, neutrophil-to-lymphocyte ratio; INR, International Normalized Ratio; IQR, interquartile range.

## 4. Discussion

### 4.1. Key Findings

This retrospective, single-center cohort study investigated the prevalence and impact of malnutrition risk among ICU patients diagnosed with either stroke (ischemic or hemorrhagic) or TBI over a period of nearly six years. The study revealed that elevated malnutrition risk was highly prevalent across all patient groups, with over 60% of patients with TBI and ischemic stroke, and over 70% of patients with hemorrhagic stroke, showing severe risk of malnutrition upon admission. Stroke patients, particularly those with hemorrhagic stroke, had a higher risk of malnutrition compared to TBI patients.

This study identified several adverse outcomes associated with malnutrition risk. Patients at risk of malnutrition exhibited higher hospital mortality rates, increased need for mechanical ventilation, and more frequent use of vasoactive drugs. Key risk factors for malnutrition included advanced age, higher SOFA scores, lower FOUR, GCS, and CRS-R scores, and the presence of pneumonia at admission. Additionally, female sex was a significant risk factor in hemorrhagic stroke patients, while coronary artery disease and arterial hypertension were notable risk factors across different patient groups.

### 4.2. Relationship with Previous Studies

The results of our study align with existing research on malnutrition in stroke patients. Previous reviews report malnutrition rates in stroke patients ranging from 6.1% to 62% [23,24]. This variability can be attributed to differences in stroke type (ischemic/hemorrhagic), comorbidities, and complications. However, the primary reason for this variability is the use of different methods and scales to assess nutritional status [17,25]. Studies utilizing the PNI have reported moderate to severe malnutrition risk rates ranging from 2% to 31% in stroke patients [24], which contrasts with our center’s higher rates of approximately 75–85%. This discrepancy likely arises because our patients were predominantly transferred to us from other centers, where they had also been in ICUs. The prolonged critical condition, combined with the accumulating negative effects of intensive therapy methods, most likely led to a systemic inflammatory response followed by the development of hypercatabolism and immunosuppression. Consequently, this resulted in low plasma albumin levels and a reduced absolute lymphocyte count, which, according to current understanding, constitutes malnutrition. Moreover, the use of common scales such as the Malnutrition Universal Screening Tool (MUST), Nutritional Risk Screening 2002 (NRS-2002), and Mini Nutritional Assessment Short Form (MNA-SF) is not feasible in our setting due to impaired consciousness and limitations in our study cohort [24,26].

Our study also confirmed that risk factors such as advanced age, reduced level of consciousness, female sex, arterial hypertension, and pneumonia are significant predictors of malnutrition risk in stroke patients [27,28]. Risk factors for malnutrition in TBI patients, such as pulmonary infection and GCS score, identified in the study by Cai et al., were also significant in our research [29]. 

One of the largest studies, the FOOD Trial Collaboration [30], investigated the impact of nutritional status on stroke patient outcomes over six years across six countries, involving 3012 patients in 112 hospitals. The study found that malnutrition significantly increased the mortality rate and the risk of complications such as pneumonia and gastrointestinal bleeding. Specifically, the mortality rate was 37% in patients at risk of malnutrition compared to 20% in those with normal nutritional status [30]. Additionally, patients with normal nutrition showed higher levels of independence in daily activities, as measured by the Rankin scale, with 30.0%, compared to 17.1% in patients at risk of malnutrition. Thus, low nutritional status in stroke patients is a predictor of poor outcomes and increased mortality. Malnutrition negatively impacts recovery, reducing motor and cognitive function recovery, social and daily adaptation, and overall quality of life [13,30,31,32].

Our analysis indicates that moderate or severe risk of malnutrition is common among TBI patients of all ages and genders. There is a clear association between the severity of malnutrition and the risk of infectious and inflammatory complications in TBI patients. Those with severe TBI are at risk for prolonged nitrogen loss and exhaustion, leading to increased susceptibility to infections, poor wound healing, and longer durations on mechanical ventilation [11,12]. Malnutrition is an independent risk factor for nosocomial infections and adverse clinical outcomes, including prolonged hospitalization and increased mortality [11,12,33]. Similarly, our study demonstrated that patients at risk of malnutrition had a higher mortality rate, longer hospital stays, and a greater frequency of mechanical ventilation use.

Our findings support previous research indicating that malnutrition adversely affects clinical outcomes and underscores the utility of PNI as a prognostic criterion [34]. For instance, S. Nergiz and U. Ozturk demonstrated a strong association between low PNI levels and secondary infections in acute stroke patients, with 21% developing secondary infections and 69% of patients experiencing malnutrition [35]. This association contributes to prolonged hospital stays and increased mortality. Our previous data also show higher malnutrition rates and hospital-acquired pneumonia incidence in early neurorehabilitation patients, attributable to longer ICU stays and the chronic nature of their conditions [36].

### 4.3. Significance of the Study Findings

The significance of our findings is supported by several key points.

Firstly, it was shown that risk of malnutrition in patients with brain injury is a risk factor for mortality, prolonged hospitalization, and the need for mechanical ventilation. It is crucial to understand that this condition may be rooted in a persistent systemic inflammatory response, leading to dysfunction in various organs and systems. This conclusion is further supported by multivariate analysis, which identifies the severity of multiple organ failure as an independent factor for mortality, and pneumonia at admission as a risk factor for malnutrition.

Secondly, our study revealed that malnutrition is not a uniform condition. Kaplan–Meier curves clearly demonstrate differences in mortality risk between patients with moderate and severe risk of malnutrition, with the PNI score being a significant predictor of mortality. This finding suggests the need for stratifying patients based on the severity of malnutrition to guide management strategies and assess the risk of adverse outcomes. Moreover, the high prevalence of severe risk of malnutrition, particularly in hemorrhagic stroke patients, underscores the need for comprehensive nutritional status assessment as a standard component of ICU care.

Thirdly, our study demonstrated that malnutrition risk is associated with profound metabolic disturbances, as evidenced by elevated NLR, increased INR, and decreased levels of albumin, total protein, and cholesterol at admission. Patients with malnutrition risk exhibited disruptions in coagulation hemostasis, immune status, and the body’s synthetic functions. This aspect underscores the high clinical significance of malnutrition, reflecting the extent of homeostatic imbalances. The study advocates treating these patients as polymorbid, emphasizing that nutritional therapy should be integrated into their broader management plans [37].

### 4.4. Strengths and Limitations

This study’s major strength lies in being conducted using a large and comprehensive EHR dataset from a single-center ICU over a six-year period.

However, several limitations should be acknowledged. Being a single-center study, the findings may not be fully generalizable to other settings with different patient demographics or healthcare practices. The retrospective design inherently carries the risk of data missingness and potential biases in data recording. The exclusion of patients with incomplete malnutrition status data or ICD-10 diagnoses might have introduced selection bias. Additionally, the observational nature of the study precludes establishing reliable causality.

Our study assessed only the risk of malnutrition using the PNI score, which incorporates serum albumin and lymphocyte count [38]. It is important to note that the PNI score may also reflect critical illness-related inflammation, which may confound its specificity for nutritional status [39]. Due to the retrospective nature of our study, we lacked the data necessary to perform physical examinations and assess muscle mass, preventing the use of the Global Leadership Initiative on Malnutrition (GLIM) criteria and other tools for malnutrition diagnosis [40]. 

Future multicenter, prospective studies are warranted to validate and expand upon these findings, incorporating comprehensive nutritional assessments including muscle mass evaluation.

## 5. Conclusions

This study demonstrates a high prevalence of severe malnutrition risk among ICU patients with traumatic brain injury, ischemic stroke, and hemorrhagic stroke, associated with worse clinical outcomes such as higher mortality and increased need for mechanical ventilation. These findings emphasize the need for early nutritional assessment and intervention to improve outcomes in ICU patients. Future multicenter studies are essential to validate and expand upon these results.

## Figures and Tables

**Table 1:** Initial parameters, disease characteristics, and outcomes of ICU patients.

Parameters		Traumatic Brain Injury (1), *n* = 267	Ischemic Stroke (2), *n* = 825	Hemorrhagic Stroke (3), *n* = 260	*p* 1–2–3	*p* 1–2 ^†^	*p* 1–3 ^†^	*p* 2–3 ^†^
Sex	male	193, 72%	449, 54%	148, 57%	<0.001 ^1^	<0.001 ^1^	<0.001 ^1^	0.5^1^
	female	74, 28%	376, 46%	112, 43%				
Age, years		42 (IQR 29–56)	69 (IQR 60–78)	59 (IQR 48–68)	<0.001 ^2^	<0.001 ^4^	<0.001 ^4^	<0.001 ^4^
BMI, kg/m^2^		* *n* = 240, 21.8 (IQR 18.9–24.9)	*n* = 744, 26.1 (IQR 23–30.6)	*n* = 240, 25.6 (IQR 22.4–29.8)	<0.001 ^2^	<0.001 ^4^	<0.001 ^4^	0.4 ^4^
Transfer from other hospital		250, 64%	803, 97%	254, 98%	0.016 ^3^	0.007 ^3^	0.031 ^3^	0.9 ^3^
APACHE II at admission, score		*n* = 15, 6 (IQR 3–10)	*n* = 28, 7.5 (IQR 5–13.5)	*n* = 10, 8 (IQR 6–14)	0.4 ^2^	-	-	-
SOFA at admission, score		*n* = 182, 3 (IQR 2–4)	*n* = 599, 3 (IQR 1–5)	*n* = 181, 3 (IQR 1–4)	0.111 ^2^	-	-	-
FOUR at admission, score		*n* = 189, 14 (IQR 11–16)	*n* = 621, 16 (IQR 13–16)	*n* = 191, 15 (IQR 12–16)	<0.001 ^2^	<0.001 ^4^	0.1 ^4^	0.7 ^4^
GCS at admission, score		*n* = 192, 11 (IQR 9–14)	*n* = 635, 14 (IQR 10–15)	*n* = 195, 14 (IQR 10–15)	<0.001 ^2^	<0.001 ^4^	<0.001 ^4^	0.9 ^4^
CRS-R at admission, score		*n* = 128, 11 (IQR 6–19)	*n* = 393, 20 (IQR 11–23)	*n* = 121, 18 (IQR 8–22)	<0.001 ^2^	<0.001 ^4^	0.017 ^4^	0.042 ^4^
Pneumonia at admission		88, 33%	292, 35%	89, 34%	0.8 ^1^	-	-	-
Comorbidity								
Coronary artery disease		17, 6.4%	213, 26%	44, 17%	<0.001 ^3^	<0.001 ^1^	<0.001 ^1^	0.003 ^1^
Arterial hypertension		82, 31%	633, 77%	197, 76%	<0.001 ^1^	<0.001 ^1^	<0.001 ^1^	0.8 ^1^
Type 2 diabetes		3, 1.1%	52, 6.3%	11, 4.2%	<0.001 ^3^	<0.001 ^3^	0.031 ^3^	0.3 ^3^
Anemia		17, 6.4%	20, 1.4%	13, 5.0%	0.005 ^3^	0.005 ^3^	0.6 ^3^	0.06 ^3^
Laboratory parameters and malnutrition risk assessment at admission								
PNI score		33.4 (IQR 28.2–37.7)	32.2 (IQR 27.3–36.7)	31.3 (IQR 27.9–35.3)	0.025 ^2^	0.1 ^4^	0.016 ^4^	0.7 ^4^
Status	Normal	60, 23%	159, 20%	32, 12%	0.012 ^1^	0.4 ^1^	0.002 ^1^	0.014 ^1^
	Moderate	47, 18%	135, 16%	37, 14%				
	Severe	160, 60%	531, 64%	191, 74%				
	WBC, 10^9^/L	8.2 (IQR 6.6–11)	8.8 (IQR 6.8–11.5)	8.6 (IQR 6.5–11.6)	0.127 ^2^	-	-	-
Lymphocyte count, 10^9^/L		1.4 (IQR 1.1–1.8)	1.2 (IQR 0.9–1.8)	1.2 (IQR 0.9–1.8)	0.005 ^2^	0.004 ^4^	0.1 ^4^	0.9 ^4^
Neutrophil count, 10^9^/L		5.6 (IQR 4.3–8.4)	6.4 (IQR 4.5–9.2)	6.4 (IQR 4.5–8.9)	0.027 ^2^	0.024 ^4^	0.1 ^4^	0.9 ^4^
NLR		4.3 (IQR 2.6–6.9)	5 (IQR 3.1–9)	5 (IQR 2.9–8.7)	<0.001 ^2^	0.001 ^4^	0.02 ^4^	0.9 ^4^
Platelets, 10^9^/L		337 (IQR 251–429)	255 (IQR 201–328)	305 (IQR 228.5–379.5)	<0.001 ^2^	<0.001 ^4^	0.01 ^4^	<0.001 ^4^
INR		*n* = 262, 1.18 (IQR 1.1–1.3)	*n* = 816, 1.15 (IQR 1.1–1.3)	*n* = 258, 1.15 (IQR 1.1–1.3)	0.026 ^2^	0.02 ^4^	0.4 ^4^	0.9 ^4^
Albumin, g/L		33.4 (IQR 28.2–37.7)	32.2 (IQR 27.3–36.7)	31.3 (IQR 27.9–35.3)	0.025 ^2^	0.1 ^4^	0.025 ^4^	0.7 ^4^
Total protein, g/L		*n* = 264, 65.1 (IQR 58.5–69.7)	*n* = 795, 62 (IQR 56.1–67.6)	*n* = 254, 61.8 (IQR 56.2–66.3)	<0.001 ^2^	<0.001 ^4^	<0.001 ^4^	0.9 ^4^
Cholesterol, mmol/L		*n* = 16, 4.9 (IQR 4.4–6.3)	*n* = 64, 3.9 (IQR 3.1–4.7)	*n* = 23, 4.4 (IQR 3.1–5.6)	0.009 ^2^	0.008 ^4^	0.3 ^4^	0.7 ^4^
Outcomes								
Hospital mortality		18, 6.7%	122, 14.8%	21, 8.1%	<0.001 ^3^	<0.001 ^3^	0.6 ^3^	0.004 ^3^
Hospital length of stay, days		39 (IQR 24–63)	31 (IQR 22–47)	36 (IQR 24–56)	<0.001 ^2^	<0.001 ^4^	0.9 ^4^	0.001 ^4^
Need for MV		126, 47%	360, 44%	137, 53%	0.035 ^1^	0.3 ^1^	0.2 ^1^	0.011 ^1^
Use of vasoactive drugs		44, 17%	144, 18%	34, 13%	0.3 ^1^	-	-	-
Discharge department	ICU palliative psych. ward neurorehabilitation	126, 43%	386, 47%	130, 50%	0.5 ^1^	-	-	-
		69, 26%	209, 25%	72, 28%				
		72, 27%	230, 28%	58, 22%				

1—Chi-square test; 2—Kruskal–Wallis test; 3—Fisher’s exact test (Fisher–Freeman–Halton exact test); 4—Mann–Whitney U-test. * Number of patients (*n*) is given when missing data are present. ^†^ Bonferroni multiple-significance-test correction applied (statistical significance set at *p* < 0.017). Abbreviations: ICU, intensive care unit; BMI, body mass index; APACHE II, acute physiology and chronic health evaluation II; SOFA, Sequential Organ Failure Assessment; FOUR, Full Outline of UnResponsiveness; GCS, Glasgow coma scale; CRS-R, Coma Recovery Scale-Revised; MV, mechanical ventilation; PNI, prognostic nutritional index; WBC, white blood cell count; NLR, neutrophil-to-lymphocyte ratio; INR, International Normalized Ratio; IQR, interquartile range.

**Table 2:** Cox regression analysis (outcome of hospital mortality).

Parameters	Univariate Analysis HR (95% CI)	*p* Value	Multivariable Analysis adj. HR (95% CI)	*p* Value
Sex (ref. male)	1.59 (1.16–2.16)	0.003		0.8
Age *	1.044 (1.032–1.055)	<0.001	1.038 (1.023–1.052)	<0.001
SOFA *	1.37 (1.30–1.45)	<0.001	1.33 (1.26–1.41)	<0.001
FOUR	0.85 (0.82–0.89)	<0.001		0.4
GCS	0.85 (0.81–0.90)	<0.001		0.2
CRS-R	0.94 (0.91–0.97)	<0.001		
Pneumonia at admission	2.17(1.59–2.96)	<0.001		0.3
Coronary artery disease	2.23 (1.58–3.14)	<0.001		0.1
Arterial hypertension	1.84 (1.28–2.64)	<0.001		0.9
Type 2 diabetes	2.08 (1.12–3.86)	0.020		0.9
Anemia	0.67 (0.21–2.11)	0.5		-
Ischemic stroke (ref. TBI)	3.06 (1.85–5.04)	<0.001		0.4
Hemorrhagic stroke (ref. TBI)	1.45 (0.77–2.73)	0.3		-
PNI score	0.918 (0.893–0.943)	<0.001		0.8

* Remained in the final model (backward stepwise (Wald) method, step 12). Abbreviations: SOFA, Sequential Organ Failure Assessment; FOUR, Full Outline of UnResponsiveness; GCS, Glasgow coma scale; CRS-R, Coma Recovery Scale-Revised; PNI, prognostic nutritional index; HR, hazard ratio; CI, confidence interval.

## Data Availability

Restrictions apply to the availability of these data. Data were obtained from RICD and are available at https://fnkcrr-database.ru (accessed on 20 June 2024) with the permission of Federal Research and Clinical Center of Intensive Care Medicine and Rehabilitology.

## Supplementary Materials

The following supporting information can be downloaded at: https://www.mdpi.com/article/10.3390/nu16152396/s1, Table S1: STROBE checklist.
